# Optical Sensor for Scanning Angle of Micromirror with Improved 2D Calibration Method

**DOI:** 10.3390/mi16091046

**Published:** 2025-09-13

**Authors:** Longqi Ran, Zhongrui Ma, Ting Li, Jiangbo He, Jiahao Wu, Wu Zhou

**Affiliations:** 1 School of Mechanical and Electrical Engineering, University of Electronic Science and Technology of China, Chengdu 611731, China; ranlongqi7@163.com (L.R.); cqlgmzr@163.com (Z.M.); li_ting25@163.com (T.L.); 2 School of Mechanical Engineering, Xihua University, Chengdu 610039, China; hejiangbo@foxmail.com; 3 Huawei Technologies Co., Ltd., Shenzhen 518129, China; wujiahao7@huawei.com

**Keywords:** optical sensors, micromirrors, rotation measurement, calibration

## Abstract

The optical angle sensor demonstrates considerable potential to supersede the piezoresistive sensor as the preferred angle detection mechanism for micromirrors, primarily due to its reduced vulnerability to temperature fluctuations. However, this sensor is susceptible to interference from rotations about non-detectable axes and exhibits inadequate linearity. To mitigate these challenges, this paper introduces a sub-region calibration method. A mapping surface was created to link the output signal offsets of two axes with their input angles, allowing the effects of non-measured axes to be treated as variables. To simplify the mathematical model of this mapping surface, it was divided into an n-by-n grid of small areas. Each area uses bilinear interpolation to calculate the corresponding angle from the output values. To quickly locate which grid area a sensor output belongs to, the entire mapping surface was scaled to a range from 0 to n. Sensor outputs are then assigned to specific grid areas using the floor function. For validation, an optical sensor and a 2D rotating stage were built for calibration tests. Experimental results show that this calibration method keeps measurement errors below 0.01° within a ±8° operating range of the sensor.

## 1. Introduction

LiDAR (Light Detection and Ranging) has emerged as a pivotal technology for autonomous vehicles, offering high-resolution and long-range detection capabilities [1,2,3]. Among its core components, two-dimensional micromirrors stand out as compact and cost-effective scanning solutions, enabling precise beam steering in LiDAR systems [4,5,6]. The operational accuracy of these micromirrors directly determines the spatial resolution and object positioning fidelity of LiDAR, as even minor torsional deviations can propagate into significant measurement errors [7,8,9]. However, environmental perturbations, including thermal fluctuations, humidity variations, and mechanical stresses, introduce nonlinear distortions in micromirror dynamics, thereby degrading system reliability [10]. To mitigate these effects, real-time closed-loop control systems incorporating high-precision angle sensors have become essential for maintaining micromirror positioning accuracy [11,12].

Current micromirror angle sensing methodologies can be classified into four primary categories: capacitive, piezoresistive, piezoelectric, and optical techniques. Capacitive sensing relies on detecting capacitance variations between electrode pairs [13], yet its angular detection range is fundamentally limited by electrode geometry, rendering it impractical for large rotation angles (>5°). Piezoresistive sensors, fabricated directly on silicon torsion beams, exploit the piezoresistive effect to infer angular displacement from beam strain [11,14,15,16]. While offering direct integration advantages, this approach suffers from temperature sensitivity and inter-axis crosstalk, particularly between fast and slow scanning axes. Piezoelectric alternatives utilize voltage generation from beam deformation via the piezoelectric effect [17], though material hysteresis and long-term stability concerns persist.

In contrast, optical sensing techniques demonstrate superior potential for non-contact, wide-range angular measurement. These systems typically employ a collimated light source and quadrant photodiode (QPD) arrays to track reflected beam displacement, with angular resolution determined by the geometric relationship between spot displacement and mirror rotation [18]. Early implementations by Ishikawa et al. achieved ±2.5° detection using vertical-cavity surface-emitting lasers (VCSELs), though with limited linearity [18]. Subsequent research has predominantly focused on displacement measurement optimization [19,20,21,22], exemplified by Inokuchi et al.’s 0.5% accuracy over 160 µm displacement [19] and Zhan et al.’s 150 nm resolution within 500 µm range [22]. Notably, angular detection capabilities in these studies remained constrained (±2° range with 0.1° accuracy), highlighting a critical performance gap between displacement and angular sensing.

Recent advancements in algorithmic compensation have partially addressed inherent nonlinearities in optical sensing. Chen et al. demonstrated accuracy improvements through higher-order polynomial fitting of photodiode responses [23], while Diao et al. implemented Bayesian calibration to achieve 15th-order equivalent precision with reduced computational load [24]. Wu et al. further enhanced robustness against spot size variations using Boltzmann function-assisted finite integral methods [25]. Despite these innovations, two persistent challenges hinder practical implementation: signal nonlinearity escalates exponentially with detection range expansion, and mechanical misalignment during packaging induces inter-axis crosstalk, causing systematic deviations between theoretical predictions and experimental results [26,27]. Although Hung Ng et al. recently achieved dual-axis detection with ±10° range and 0.0265° accuracy [27], their system required complex calibration protocols that undermine scalability.

While incremental progress has been made in both detection range and accuracy over the past decade, fundamental limitations persist. Existing optical sensors struggle to reconcile wide angular ranges (>±10°) with sub-0.1° accuracy, particularly when accounting for environmental disturbances and inter-axis interference. This work addresses these challenges through a novel optical sensing architecture combining adaptive spot shaping and crosstalk-decoupled signal processing, aiming to advance micromirror control for next-generation LiDAR systems.

This study presents an optical-based angle detection methodology for 2D micromirror systems, accompanied by a novel calibration algorithm designed to address two critical error sources: nonlinearity in sensor response and measurement crosstalk arising from the coupled rotation of dual axes. The proposed solution implements a segmented calibration approach that partitions the relationship between sensor output signals and angular displacement into discrete operational regions. Within each partitioned region, a bilinear interpolation computational model is applied to establish localized linear approximations. This architecture achieves a huge reduction in computational complexity compared to high order polynomial calibration methods. Experimental validation demonstrates an improvement in angle measurement accuracy through the implementation of the proposed calibration framework, with residual errors maintained below 0.01° across ± 8° operational ranges of both axes.

## 2. Design and Calibration of the Optical Angle Sensor

### 2.1. System Assembly

**Figure 1:**
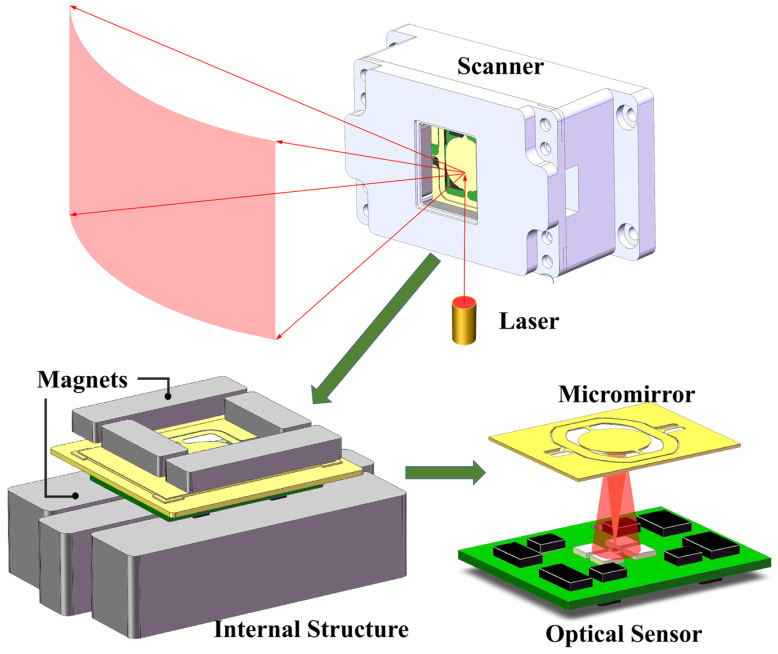
Micromirror and optical angle sensor module.

Figure 1 illustrates the schematic configuration of the packaged system. The 2D magnetic micromirror with a maximum mechanical rotation angle of ±7.5° is positioned at the system’s apex to direct the laser reflection. The electromagnetic dual-axis micromirror consisted of a reflecting mirror, a wire coil, a wire frame, and four torsion beams. The 8 mm reflective mirror and the coil frame were connected by two beams, referred to as the fast-scanning axis, while the beams linking the coil frame to the outer frame of the micromirror formed the slow-scanning axis. Below the micromirror resides the optical angle sensor module, comprising a central LED encircled by four PDs for mirror tilt angle detection. The sensor-emitted laser beam reflects from the rear surface of the mirror and is captured by the PD array. The tilting angle of the mirror induces relative motion between the micromirror and sensor, thereby modulating the reflected laser intensity. These intensity variations are detected by the PDs, enabling high-precision angular displacement measurement. Permanent magnets are placed to generate the magnetic field required for the micromirror.

**Figure 2:**
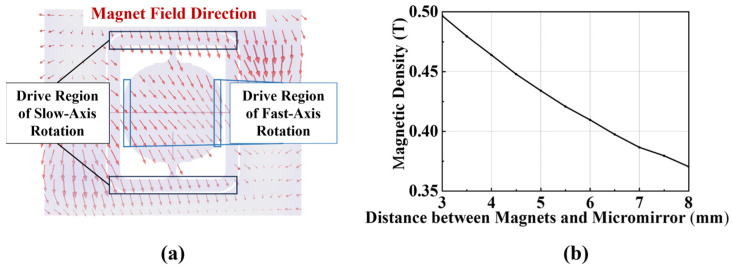
Magnetic field analysis of the micromirror system. (**a**) Magnetic field distribution around the micromirror; (**b**) Magnetic field variations in the slow-axis actuation region with increasing vertical distance between the magnet and micromirror.

To achieve sufficient rotation angles for the micromirror, the device requires exposure to a strong magnetic field, which necessitates minimal separation between the micromirror and the optical sensor. Finite element simulations were conducted to analyze the magnetic field distribution around the micromirror (see Figure 2a). Figure 2b demonstrates the magnetic field variations in the slow-axis actuation region as the vertical distance between the magnet and micromirror increases. To maintain adequate magnetic field strength for actuation, the magnet-to-micromirror separation must be ≤0.5 mm.

### 2.2. Working Principle of Optical Angle Sensor

**Figure 3:**
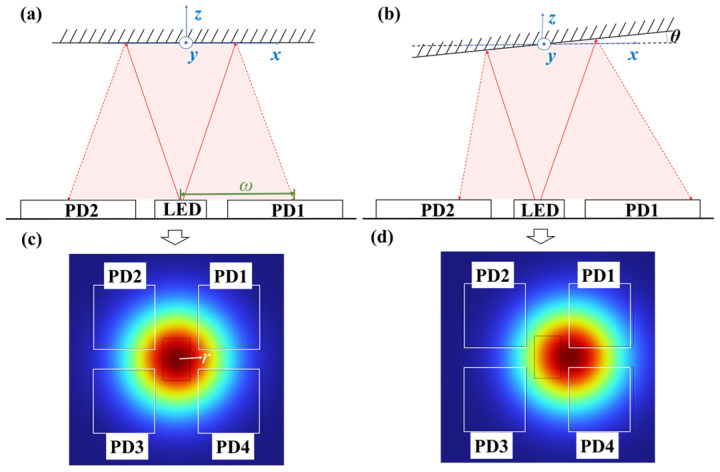
Schematic diagram of the detection principle. (**a**) The path of the reflected light when the mirror has no rotation; (**b**) The path of the reflected light when the mirror rotates by an angle of *θ* along the y-axis; (**c**) Position of the reflected light spot when the mirror has no rotation; (**d**) Position of the reflected light spot when the mirror rotates by an angle of *θ* along the y-axis.

The detection principle for *x*-axis and *y*-axis angle measurements using the optical sensor is illustrated in Figure 3. The system employs a Gaussian beam propagating along the *z*-axis, with intensity distribution described by

(1) $$I=\frac{2P_{0}}{\pi \omega^{2}}\exp\left(-2\frac{r^{2}}{\omega^{2}}\right)$$

where *P*_0_ represents total beam power, ω denotes transmitter aperture, ω indicates beam half-width, and *r* is the radial distance from the beam center. Surface tilt induces lateral displacement of the reflected light spot, as shown in Figure 3c,d, creating differential intensity variations across the PD arrays. For anticlockwise *x*-axis rotation, PD1 and PD2 exhibit intensity increases while PD3 and PD4 show decreases. Conversely, *y*-axis anticlockwise rotation produces intensity increases in PD1 and PD4 with corresponding decreases in PD2 and PD3.

**Figure 4:**
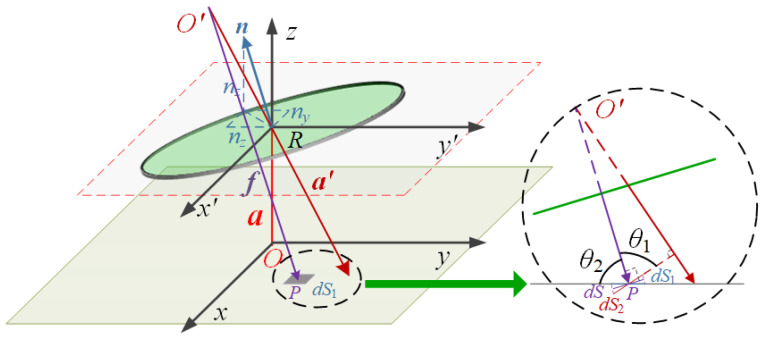
Schematic diagram of geometric principle analysis for the optical angle sensor.

The 2D rotation angle detection of the optical sensor is described in the *x-y-z* coordinate system (see Figure 4). The light source with beam divergence half angle *θ_beam_* is positioned at the origin, O (0, 0, 0). The mirror is placed above the light source at distance h and rotates along the *x*-axis and *y*-axis by angles *θ_x_* and *θ_y_*, respectively. As a result, the normal vector of the mirror is expressed as

(2) $$n={\left(C_{y}C_{x}\right)}^{T}\left[\begin{array}{c} 0\\ 0\\ 1 \end{array}\right]=\left[\begin{array}{c} \sin\theta_{y}\\ -\cos\theta_{y}\sin\theta_{x}\\ \cos\theta_{y}\cos\theta_{x} \end{array}\right]=\left[\begin{array}{c} n_{x}\\ n_{y}\\ n_{z} \end{array}\right]$$

where **C_x_** and **C_y_** are the rotation matrices of the mirror and are expressed as

(3) $$C_{x}=\left[\begin{array}{ccc} 1 & 0 & 0\\ 0 & \cos\theta_{x} & \sin\theta_{x}\\ 0 & -\sin\theta_{x} & \cos\theta_{x} \end{array}\right],C_{y}=\left[\begin{array}{ccc} \cos\theta_{y} & 0 & -\sin\theta_{y}\\ 0 & 1 & 0\\ \sin\theta_{y} & 0 & \cos\theta_{y} \end{array}\right]$$

The illumination of the reflected light on the *x-y* plane can be regarded as the direct illumination from the light source onto the mirror point on the plane. The coordinates of the mirror point illuminated by the light source can be calculated as

(4) $$O^{\prime }=2\left(a\cdot n\right)n=\left[\begin{array}{c} 2hn_{x}n_{z}\\ 2hn_{y}n_{z}\\ 2hn_{z}n_{z} \end{array}\right]$$

where ***n*** is the normal vector of the mirror. And the vector of the reflected beam can be calculated as ***a′*** (−2*hn_x_n_z_*, −2*hn_y_n_z_*, *h*−2*hn_z_n_z_*).

The height of the PDs is *h_p_*. Since the reflected beam does not illuminate the photodiode vertically, the intensity received by the PDs can be calculated by integrating intensity element *dS*. Based on (1), *l* is the projection of f onto the vector *a′* and *r* is the perpendicular distance from *dS* to *a’*, which are required to calculate the intensity. According to the geometric relation, *l* and *r* can be derived as

(5) $$l=f\cdot n_{A}=f\cdot \frac{a^{\prime }}{\left|a^{\prime }\right|}=\frac{\left|2n_{z}n_{x}\left(2hn_{z}n_{x}-x\right)+2n_{z}n_{y}\left(2hn_{z}n_{y}-y\right)+\left(1-2n_{z}n_{z}\right)\left(2hn_{z}n_{z}-h_{p}\right)\right|}{\sqrt{{\left(2n_{z}n_{x}\right)}^{2}+{\left(2n_{z}n_{y}\right)}^{2}+{\left(1-2n_{z}n_{z}\right)}^{2}}}$$

(6) $$r=\sqrt{{\left|f\right|}^{2}-l^{2}}$$

And ω can be derived as

(7) $$\omega =l\tan\theta_{beam}$$

By substituting Equations (6) and (7) into Equation (1), the illuminance of the infinitesimal element ds can be obtained. So, the optical power received by the PDs can be calculated as

(8) $$P=\int I_{1}dS_{1}=\int I\frac{\sin\theta_{1}}{\sin\theta_{2}}dS_{2}$$

Photocurrents from the PDs undergo voltage conversion for signal processing, with output voltages *u*_1_, *u*_2_, *u*_3_, and *u*_4_ corresponding to light intensities. Previous research [22,24,25] established angular relationships through output signal offset (OSO) parameters, which are defined for *x*-axis and *y*-axis measurements as

(9) $$\left\{\begin{array}{l} \sigma_{x}=\frac{\left(u_{1}+u_{2}\right)-\left(u_{3}+u_{4}\right)}{u_{1}+u_{2}+u_{3}+u_{4}}\\ \sigma_{y}=\frac{\left(u_{1}+u_{4}\right)-\left(u_{2}+u_{3}\right)}{u_{1}+u_{2}+u_{3}+u_{4}} \end{array}\right.$$

**Figure 5:**
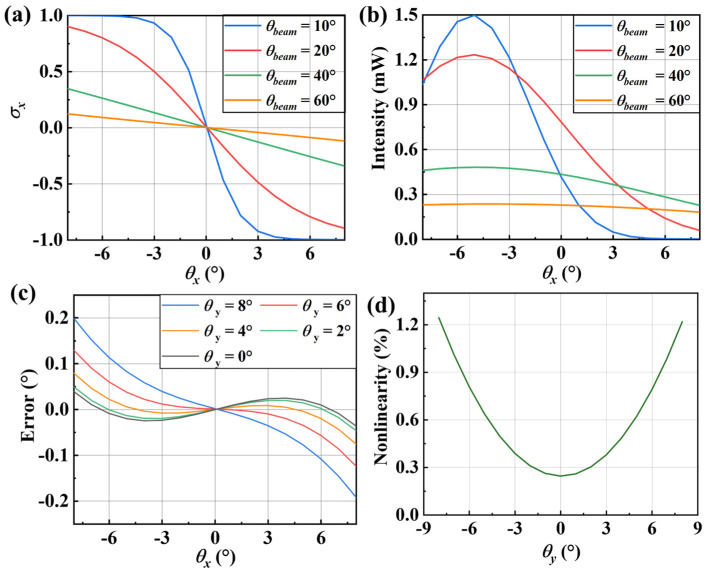
Simulation results. (**a**) Output signal offsets under different beam divergence angles. (**b**) Light intensity versus tilting angle under different beam divergence angles. (**c**) The error in detecting the tilt angle around the *x*-axis under different *y*-axis tilt angles. (**d**) The nonlinearity in detecting the tilt angle around the *x*-axis under different *y*-axis tilt angles.

The simulation data were substituted into (9), and the corresponding results are shown in Figure 5. The influence of the laser beam divergence angle on the output is calculated (see Figure 5a,b) based on the analysis described above. As the divergence angle increases, while maintaining the same laser power, the linearity of the output signal offset improves. Although the linearity of the output signal offset improves as the divergence angle increases while maintaining the same laser power, the received light intensity variation for a single PD decrease, which reduces the signal-to-noise ratio and thereby hinders high-precision detection.

Notably, even when maintaining identical tilt angles *θ_x_* about the *x*-axis on the measured surface, sensor output *σ_x_* remains susceptible to interference from tilt angles *θ_y_* about the *y*-axis. Furthermore, the sensor output exhibits a notable overall nonlinearity of 0.1%. To address these compounded errors, we propose a dual-input mapping framework where OSO parameters (*σ_x_*, *σ_y_*) serve as independent variables predicting tilt angles (*θ_x_*, *θ_y_*) as dependent variables, as illustrated in Figure 5c,d.

### 2.3. Calibration Method

The direct implementation of complex mapping from (*σ_x_*, *σ_y_*) to (*θ_x_*, *θ_y_*) proves computationally prohibitive for practical hardware, rendering practical implementation challenging. To mitigate computational complexity, the mapping surface connecting the output signal to tilt angles is divided into multiple sub-regions, with linear interpolation functions subsequently applied within each partitioned region to approximate the input–output relationship. This approach involves two critical steps: the strategic partitioning of the mapping surface combined with output localization to corresponding regions and the functions in each region.

To systematically partition the mapping surface into an *n* × *n* grid of regions and enable precise data localization, spatial quantization begins with output scaling transformation:

(10) $$\left\{\begin{array}{l} \delta_{x}=n\left[\sigma_{x}-\min\left(\sigma_{x}\right)\right]/\left[\max\left(\sigma_{x}\right)-\min\left(\sigma_{x}\right)\right]\\ \delta_{y}=n\left[\sigma_{y}-\min\left(\sigma_{y}\right)\right]/\left[\max\left(\sigma_{y}\right)-\min\left(\sigma_{y}\right)\right] \end{array}\right.$$

This normalization OSO values to [0, *n*] range, with each sub-region having a unit side length of 1. This configuration establishes a coordinate system where each sub-region can be uniquely identified through the integer coordinates of its vertices. To determine the corresponding sub-region, the floor function is applied to the rescaled OSOs *δ_x_* and *δ_y_*, effectively mapping them to discrete integer indices. Discrete coordinate indexing via floor function operation

(11) $$\left(i,j\right)=\left\lfloor \delta_{x},\delta_{y}\right\rfloor$$

establishes integer vertex coordinates (*i*, *j*), with adjacent nodes at (*i* + 1, *j*), (*i*, *j* + 1) and (*i* + 1, *j* + 1). Here, the symbol ⌊⌋ indicates rounding down.

**Figure 6:**
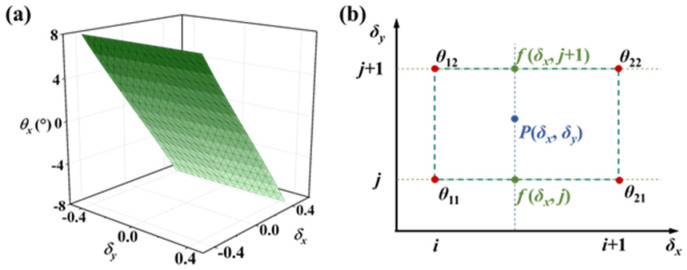
(**a**) The mapping surface between (*σ_x_*, σ*_y_*) and *θ_x_*; (**b**) description of bilinear interpolation method.

Another critical aspect lies in the interpolation scheme within grid cells. Since the four vertices of a grid cell may not necessarily be coplanar, bilinear interpolation within grid cells addresses non-planar vertex configurations. Taking tilt angle *θ_x_* as an example, consider a region cell with four vertices: *Q*_11_ = (*i*, *j*), *Q*_12_ = (*i*, *j* + 1), *Q*_21_ = (*i* + 1, *j*), and *Q*_22_ = (*i* + 1, *j* + 1) (see Figure 6b). The interpolation process consists of two sequential linear interpolations. First, *δ_x_* interpolation yields

(12) $$\begin{array}{l} f\left(\delta_{x},j\right)=\frac{\left(i+1\right)-\delta_{x}}{\left(i+1\right)-i}\theta_{11}+\frac{\delta_{x}-i}{\left(i+1\right)-i}\theta_{21}\\ f\left(\delta_{x},j+1\right)=\frac{\left(i+1\right)-\delta_{x}}{\left(i+1\right)-i}\theta_{12}+\frac{\delta_{x}-i}{\left(i+1\right)-i}\theta_{22} \end{array}$$

Subsequently, linear interpolation is performed in the *δ_y_* direction, yielding

(13) $$\begin{array}{cc} f\left(P\right) & =\frac{\left(j+1\right)-\delta_{y}}{\left(j+1\right)-j}f\left(\delta_{x},j\right)+\frac{\delta_{y}-j}{\left(j+1\right)-j}f\left(\delta_{x},j+1\right)\\ & =\left(i+1-\delta_{x}\right)\left(j+1-\delta_{y}\right)\theta_{11}+\left(\delta_{x}-i\right)\left(j+1-\delta_{y}\right)\theta_{21}\\ & +\left(i+1-x\right)\left(y-j\right)\theta_{12}+\left(x-i\right)\left(y-j\right)\theta_{22} \end{array}$$

This approach does not include higher-order terms, thereby reducing the computational resource requirements.

## 3. Experiments

**Figure 7:**
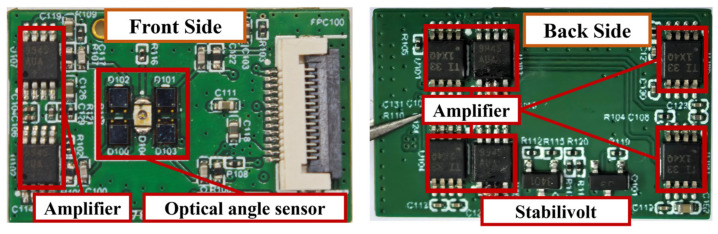
Optical angle sensor module.

Figure 7 details the angle sensor module configuration. The central LED (APTD2012F3C, Kingbright, Taiwan, China) emits 940 nm infrared radiation with a power of 8 mW and has a 40° divergence angle (defined as the angular deviation from the optical axis where luminous intensity reduces to half the peak value). Four symmetrically arranged PDs (LSSPD-2.5, LightSensing, Beijing, China) with a dimension of 2.5 mm × 2.5 mm and a responsivity of 0.6 mA/mW detect infrared radiation reflected from the tilting micromirror.

**Figure 8:**
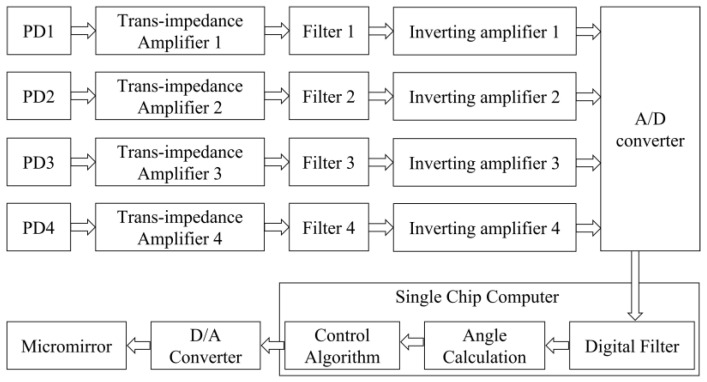
Circuit diagram of the system.

The electronic circuitry of the sensor module is presented in Figure 8. The PDs output current signals with a response time of 5 ns. Then, the current signals are initially converted to voltage signals via transimpedance amplifiers built with an operational amplifier (MAX9945AUA+, Analog Devices, Inc., Norwood, MA, USA) featuring a bandwidth of 3 MHz. Subsequent signal conditioning includes low-pass filtering and polarity inversion through inverting amplifiers composed of an operational amplifier (INA821IDGKR, Texas Instruments, Dallas, TX, USA) with a bandwidth of 4.7 MHz, yielding processed positive voltage outputs for microcontroller acquisition. Subsequently, the signal is converted into a digital signal by a high-speed ADC (AD4115, Analog Devices, Inc., Norwood, MA, USA), followed by a smoothing filter with a window size of 10 in the MCU ( STM32H7, STMicroelectronics, Geneva, Switzerland).

The response time of the system consists of four parts: the analog section, the ADC conversion time, the smoothing filter time, and arithmetic operation delay. Since the response time of the analog section is far shorter than the other two, it is no longer considered. The ADC conversion time depends only on the sampling rate, calculated as

(14) $$t_{ADC}=\frac{1}{25000\mathrm{SPS}}=40\mu s$$

For a moving average smoothing filter, the delay formula is

(15) $$t_{Filter\_delay}=\frac{\left(\mathrm{Windowsize}-1\right)t_{ADC}}{2}=180\mu s$$

With the main frequency of STM32H7 is 550 MHz, the arithmetic operation delay is negligible compared to other digital delays. So, the total response time of the device is about 220 μs.

**Figure 9:**
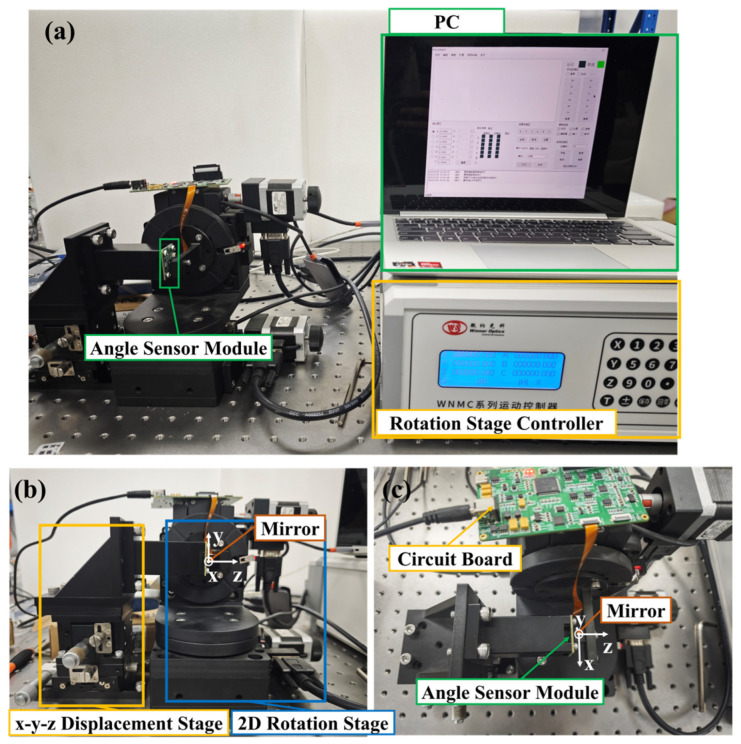
Calibration experiment environment. (**a**) Overview of the experiment environment; (**b**) Front view; (**c**) Top view.

A calibration experiment environment was built as shown in Figure 9. The system consists of an angle sensor module, a 2D rotational stage, an XYZ translational stage, a PC, and a mirror. The optical angle sensor module is mounted on the three-axis displacement stage, allowing its position to be adjusted to align with the rotation center of the 2D rotation stage. The mirror is positioned at the center of the 2D rotation stage and reflects the light emitted by the sensor’s laser.

**Figure 10:**
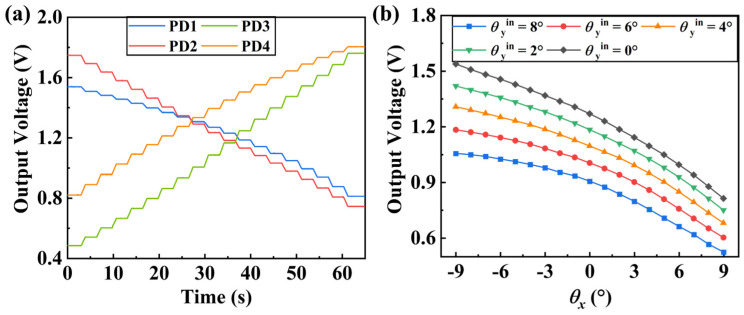
Outputs of the sensor. (**a**) Voltage of the PD output. (**b**) The OSOs about the *x*-axis under different *y*-axis tilt angles.

The two-dimensional rotating table is controlled by a PC. First, the table is rotated around the *x*-axis, followed by the rotation around the *y*-axis, with each step set to 1°. The output signals of PDs are shown in Figure 10a. As the rotation angle of the micromirror changes, the PD output signals exhibit a stepwise change. The amplifications of the four signals are not completely consistent due to the varying electrical properties of components, such as resistors, which cannot be identical.

Substituting the experimental results into Equation (9), taking the case of the tilt angle about the *x*-axis as an example, the relationship between the OSO about the *x*-axis and the tilt angle is shown in Figure 10b. As can be seen, the actual test results exhibit a higher nonlinearity compared to the theoretical results. 

**Figure 11:**
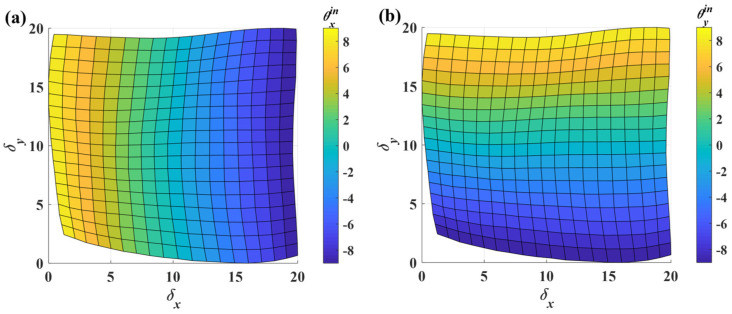
The rescaled mapping surface after rescale. (**a**) The mapping surface from (*δ_x_*, *δ_y_*) to *θ_x_*. (**b**) The mapping surface from (*δ_x_*, *δ_y_*) to *θ_y_*.

**Figure 12:**
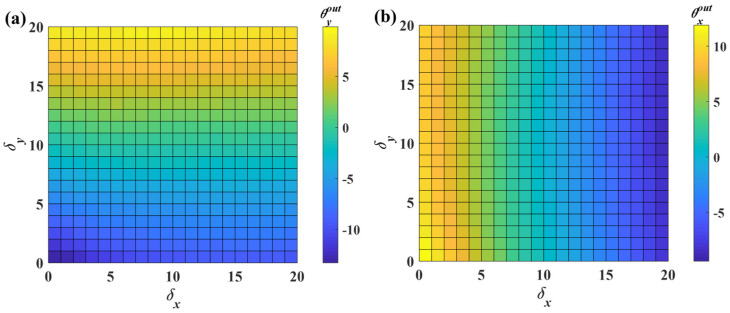
Mapping after interpolation and divided into regions. (**a**) The mapping surface from (*δ_x_*, *δ_y_*) to *θ_x_*. (**b**) The mapping surface from (*δ_x_*, *δ_y_*) to *θ_y_*.

Additionally, the tilt angle about the *y*-axis also contributes to the error. To reduce the crosstalk error and improve linearity, the following procedure applies the proposed calibration method to the sensor. Initially, the OSOs are scaled to the range of 0–20 using Equation (10). To visualize the scaled mapping surface more clearly, a two-dimensional representation is generated where color intensity corresponds to angular magnitude, as shown in Figure 11. Notably, the scaled mapping surface does not fully occupy the 0–20 output range. When the sensor inputs *δ_x_* and *δ_y_*, it is challenging to directly determine the corresponding region based on these inputs. Therefore, the entire mapping surface is reconstructed as a regular rectangular surface through interpolation using thin-plate spline interpolation [28] (see Figure 12). The detailed calculation process of thin-plate spline interpolation can be found in the Supplementary Materials.

In each region, the corresponding tilting angle and the OSO of four vertices were submitted into Equation (13). The parameters of each region can be calculated and stored in a single-chip computer. At this point, the calibration process is complete.

When the sensor performs detection, the output signal is converted into the rescaled OSO. This value is then substituted into Equation (11) and rounded down using the floor function, allowing the rescaled OSO to be positioned within the corresponding region. By invoking the parameters from the relevant region and substituting them into Equation (13), the detected angle value can be computed.

**Figure 13:**
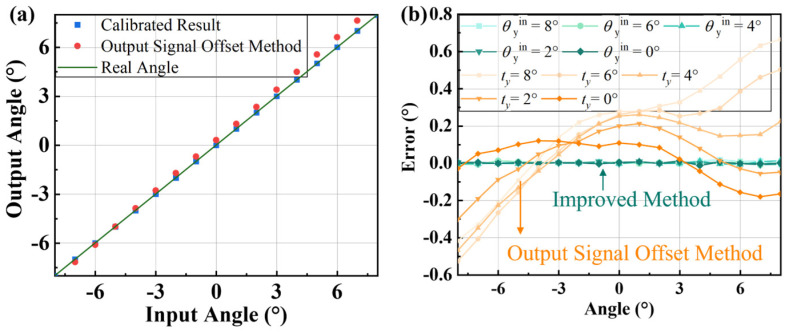
Output comparison. (**a**) The input angle versus the output angle in different calibration methods. (**b**) Comparison of output errors, where the orange represents the OSO method and the green indicates the method proposed.

To verify the calibration results, the test is carried out again in the range of −8° to 8°. At this point, a set of real angle and sensor output angle data are obtained. The output error is shown in Figure 13. Compared to the method that uses output signal offset calibration, the proposed region calibration method reduces nonlinearity from 3.75% to 0.06%. And the maximum measurement error is reduced from 0.6° to 0.01°. Moreover, it reduces the influence of the non-detection axis rotation on the error within the entire measurement range.

**Figure 14:**
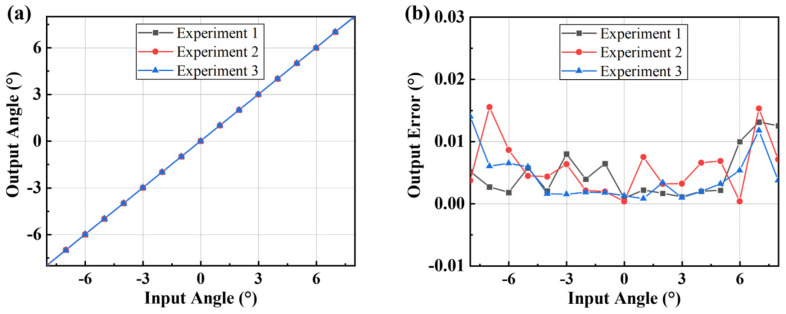
Repeated-experiments results. (**a**) Comparison of outputs; (**b**) comparison of output errors.

Repeated experiments were carried out to verify the stability of the sensor, and a set of data with the largest error caused by the rotation of the non-detection axis was selected. The results are shown in Figure 14. The output angle always maintains a good linear relationship with the input, and the maximum error is less than 0.02°, which indicates that the measurement system has excellent stability.

**Figure 15:**
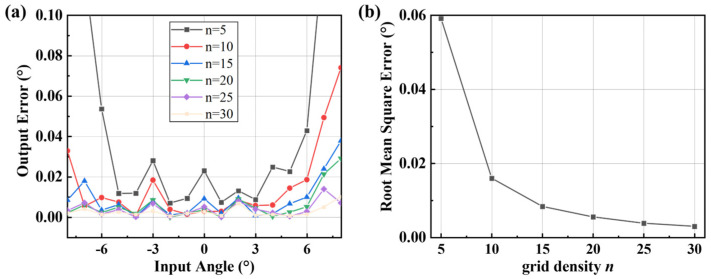
The output error under different grid densities. (**a**) Error distribution under different grid densities. (**b**) Root mean square error under different grid densities.

The impact of different grid densities on calculational errors was analyzed. Under the same rotation angle of the non-detection axis, the error distribution was presented in Figure 15a. Detection errors decrease as grid density increases, while larger rotation angles correspond to greater errors. The root mean square errors (RMSEs) under various grid densities were extracted as shown in Figure 15b. It is evident that as the number of grides increases, the RMSE exhibits an exponential downward trend. However, higher grid density drastically elevates the memory demand of the hardware. This is because, in accordance with Formula (13), each additional grid requires eight more parameters to compute the rotation angles around the *x*-axis and *y*-axis. Therefore, the selection of grid density shall be determined by the memory capacity of the hardware and the required computational precision.

## 4. Conclusions

This study aimed to address two critical challenges limiting optical angle sensors for MEMS micromirrors: biaxial crosstalk and poor linearity under large angular ranges. To resolve these issues, a sub-region calibration method was proposed: the mapping surface between the sensor’s output signal offsets and micromirror tilt angles was partitioned into an *n* × *n* grid, and bilinear interpolation was applied within each grid cell to establish localized linear approximations. This approach avoided the high computational complexity of high-order polynomial fitting while maintaining high precision. A prototype optical sensor module integrated 940 nm LED and 2.5 mm × 2.5 mm PDs were built for validation. A calibration experimental environment was built with the help of a high-precision two-dimensional rotating platform. The experimental results confirmed that within a ±8° operating range, the method reduced nonlinearity from 3.75% (the uncalibrated OSO method) to 0.06% and minimized the maximum measurement error from 0.6° to 0.01°, with a system response time about 220 μs to meet real-time control requirements. While the optical sensor is less temperature-sensitive than piezoresistive alternatives, this study did not evaluate its performance under long-term exposure to vibration, humidity, or thermal cycling, which are critical for automotive or industrial applications. Future work will integrate environmental compensation algorithms.

In summary, this study demonstrates a low-complexity, high-precision calibration method for MEMS micromirror optical angle sensors. Its quantified performance advantages over those in the existing literature, broad applicability to LiDAR and MEMS systems, and clear pathways to address current limitations position it as a valuable contribution to micro-optomechanical sensing technology.

## Data Availability

Data are available upon request via personal contact with the corresponding author via the email address zhouwu916@uestc.edu.cn.

## Supplementary Materials

The following supporting information can be downloaded at: https://www.mdpi.com/article/10.3390/mi16091046/s1, Thin Plane Spline Interpolation.
