# Supplementary material for: Optical Sensor for Scanning Angle of Micromirror with Improved 2D Calibration Method
Source: Micromachines (Basel). 2025 Sep 13;16(9):1046. doi: 10.3390/mi16091046 (PMC12471556; doi:10.3390/mi16091046)
Supplement: Supplementary file 1 [file micromachines-16-01046-s001.zip › micromachines-3831373-supplementary.pdf]

Supplementary

Thin Plate Spline (TPS) Interpolation is a meshless interpolation method. Its core idea is to simulate the "smooth surface/curve naturally formed by an elastic thin plate when it is forced to pass through all known data points", thereby realizing the continuous value prediction of unknown points.

There are two conditions of TPS function $f(\delta_x, \delta_y)$:

1) Interpolation Condition: For all known control points (δ_{xi}, δ_{yi}, θ_i), satisfied $\theta_i = f(\delta_{xi}, \delta_{yi})$;
2) Smoothness Condition: The bending energy of the function is minimized.

the 2D TPS function can be described as

$$\theta_x^{out} = f\left(\delta_x, \delta_y\right) = \sum_{i=1}^{m} \lambda_i \phi(r_i) + \left(d_{00} + d_{10}\delta_x + d_{01}\delta_y\right) \tag{S1}$$

Where the first term of this function is the spline term and the second term is the linear term. ri denotes the Euclidean distance between the point to be interpolated (δx, δy): and the i_{th} control point (δ_{xi}, δ_y), $\phi(r)$ is the radial basis function and $\phi(r) = r^2 \ln r$ here. λ, d_{00}, d_{10}, and d_{01} are the coefficients that can be obtained by solving a system of linear equations formed by the interpolation condition and smoothness condition, which can be described as:.

$$\begin{bmatrix} 0 & \phi(r_{12}) & \phi(r_{13}) & \cdots & \phi(r_{1n}) & 1 & \delta_{x1} & \delta_{y1} \\ \phi(r_{21}) & 0 & \phi(r_{23}) & \cdots & \phi(r_{2n}) & 1 & \delta_{x2} & \delta_{y2} \\ \vdots & \vdots & \vdots & \vdots & \vdots & \vdots & \vdots & \vdots \\ \phi(r_{n1}) & \phi(r_{n2}) & \phi(r_{n3}) & \cdots & 0 & 1 & \delta_{xn} & \delta_{yn} \\ 1 & 1 & 1 & \cdots & 1 & 0 & 0 & 0 \\ \delta_{x1} & \delta_{x2} & \delta_{x3} & \cdots & \delta_{xn} & 0 & 0 & 0 \\ \delta_{y1} & \delta_{y2} & \delta_{y3} & \cdots & \delta_{yn} & 0 & 0 & 0 \end{bmatrix} \begin{bmatrix} \lambda_1 \\ \lambda_2 \\ \vdots \\ \lambda_n \\ d_{00} \\ d_{10} \\ d_{01} \end{bmatrix} = \begin{bmatrix} \theta_{x1} \\ \theta_{x2} \\ \vdots \\ \theta_{xn} \\ 0 \\ 0 \\ 0 \end{bmatrix} \tag{S2}$$
